# Comparative Evaluation and Correlation of Pain Pattern in Neck Musculature Observed in Mild, Moderate, and Severe Temporomandibular Joint Disorder Cases as Compared to Non-temporomandibular Joint Disorder Cases

**DOI:** 10.7759/cureus.30099

**Published:** 2022-10-09

**Authors:** Abhishek D Sanchla, Sunita Shrivastav, Lovely Bharti, Ranjit Kamble

**Affiliations:** 1 Department of Orthodontics, Sharad Pawar Dental College and Hospital, Datta Meghe Institute of Medical Sciences, Wardha, IND

**Keywords:** helkimo index, extra-craniofacial symptoms, neck pain, temporomandibular joint, tmj disorders

## Abstract

Introduction

Temporomandibular disorder (TMD) is a broad term used to describe several interlinked disorders affecting the temporomandibular joint (TMJ), muscles of the craniofacial region, and associated structures, all of which have common symptoms such as pain and reduced jaw opening. Along with these, extra-craniofacial symptoms may also be present, which need to be assessed for early diagnosis of TMD. Considering the extra-craniofacial symptoms of TMD, this observational study aimed to evaluate the severity of pain in the neck musculature of patients with TMD and correlate it with the severity of the disorder.

Material and methods

A total of 44 patients were included in the study who were graded for severity of TMD based on the amnestic and dysfunctional components of the Helkimo index separately. The pain was evaluated bilaterally in five groups of neck muscles in all cases using manual palpation. The severity of pain in these neck muscles was then correlated with the severity of both subjective and objective symptoms of TMD and compared with patients without TMD.

Results

The Chi-square test revealed a statistically significant association between the severity of pain in all five groups of neck muscles and the severity of TMD. The severity of pain increased with the increasing severity of TMD, with a total of 59.09% of TMD patients presenting with varying degrees of pain in the neck musculature and a p-value of 0.0001% which was significant. Negligible pain in the neck musculature was present in patients without TMD.

Conclusion

Based on the results, it was observed that the severity of TMD directly correlated with pain in various groups of neck muscles. 59.09% of patients with TMD reported varying degrees of pain in the neck musculature. The severity and distribution of pain in the neck muscles increased with the increasing severity of TMD.

## Introduction

Temporomandibular disorder (TMD) is a broad term used to describe several interlinked disorders affecting the temporomandibular joint (TMJ), muscles of the craniofacial region, and associated structures, all of which have common symptoms such as pain and reduced jaw opening [1]. According to Rieder et al., 33% to 50% of patients had one of the symptoms of TMD. However, the patients that need professional attention range around 10% [2]. At the point when patients with temporomandibular disorder, or TMD, present their pain to the clinician, he/she might consider the pain source equivalent to its site. Tragically, the pain could be alluded to from a distant site [3]. This pain may be of otologic origin, neurologic origin, or may originate from the neck musculatures, which can be both a cause and a symptom of TMD [4]. Neuroanatomical and function-related associations among masticatory and cervical areas are discussed as reasons for accompanying jaw and neck symptoms [5]. Referred pain is predominant in the head and neck region and habitually confounds the specialist and the patient. Most patients experiencing these referred pain symptoms report to the general physician or otolaryngologist but not to an orthodontist; hence appropriate referral becomes essential [6].

Additionally, TMD is also reported to negatively affect body posture owing to the neuroanatomical manifestations, further aiming at a need for a multidisciplinary approach to diagnosis and management of TMD [7]. Luckily, these pain patterns are somewhat identical and comparable from one patient to another, empowering experts to know about these examples to promptly recognize the pain's source more [8-9]. Through palpation of muscle and trigger focus, Wright et al., associated identified referred pain locations for different muscles, neck muscles being the most common [3]. Most of the studies in the literature on TMD are related to malocclusion, teeth, and Orthodontic treatment. However, only a few studies have been carried out assessing the extra-craniofacial symptoms of TMD, which may help in the early diagnosis of TMD in patients seeking Orthodontic treatment and help in taking measures to stop the further progress of TMD [10]. De Laat et al. found that, on palpation, 23-67% of the patients with TMD had neck muscle tenderness in the sternocleidomastoid and upper trapezius as well as other cervical and shoulder muscles, which was only rarely present in the control group [11]. It is observed that neck muscle pain may be present in patients with TMD, so there was a need to precisely correlate the severity of neck muscle pain with the severity of TMD as no study in literature evaluated a direct correlation for the same; hence this study was planned.

## Materials and methods

Sample

This observational study was conducted in the Department of Orthodontics and Dentofacial Orthopedics, Sharad Pawar Dental College, Wardha, with approval from Datta Meghe Institute of Medical Sciences Ethical Committee (approval number DMIMS(DU)/IEC/2020-21/257). A total of 44 adult patients (18-30 years of age) were randomly selected from the outpatient department (OPD). The sample size was calculated based on the Cochran formula for sample size calculation - n= Z^2^_α/2_ ×p×(1-p)/E^2^, where Z^2^_α/2_ is the level of significance at a 95% confidence interval, p shows the prevalence of multiple pain conditions, and E is the error of margin (7%). Inclusion criteria were patients with permanent dentition above 18 years of age having a class I or class II (vertical) skeletal pattern. Exclusion criteria were patients with a history of Orthodontic treatment, psychological disorders, history of trauma to TMJ or TMJ surgery, and patients with bony disorders.

**Figure 1:**
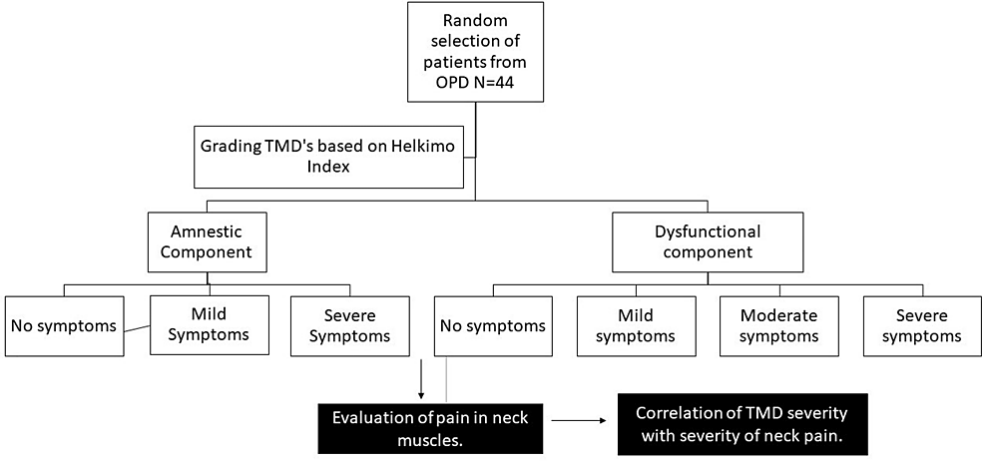
Outline of the study design OPD: outpatient department, TMD: temporomandibular disorder

They were categorized into four groups based on the dysfunctional component (objective symptoms) of the Helkimo index: group one - Patients with mild TMJ dysfunction, group two - Patients with moderate TMJ dysfunction, group three - Patients with severe TMJ dysfunction, group four - Patients with no TMJ dysfunction. Simultaneously based on the amnestic component of the Helkimo index, subjective symptoms in the same patients were also graded into no, mild and severe varieties. The study design is depicted in Figure 1.

Methods

**Figure 2:**
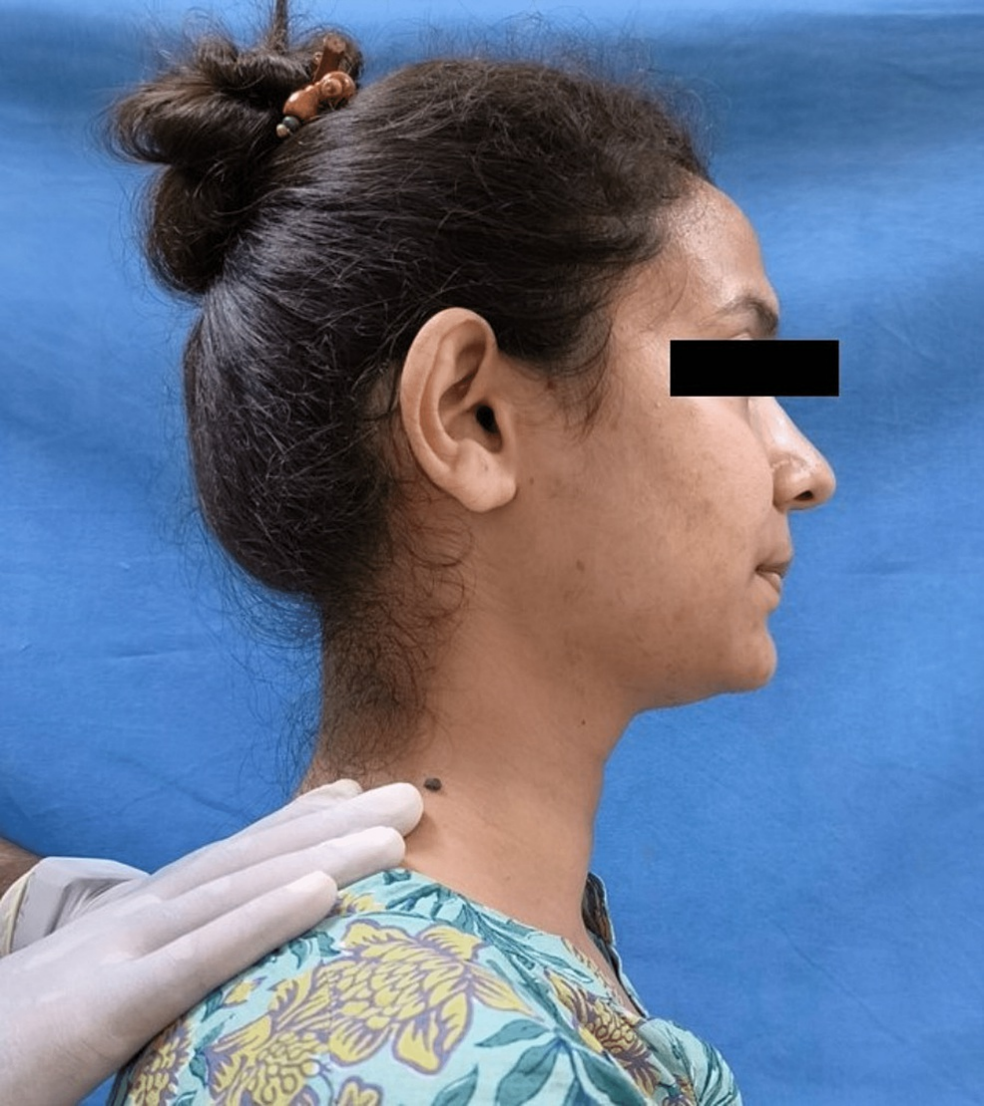
Palpation of the trapezius muscle

**Figure 3:**
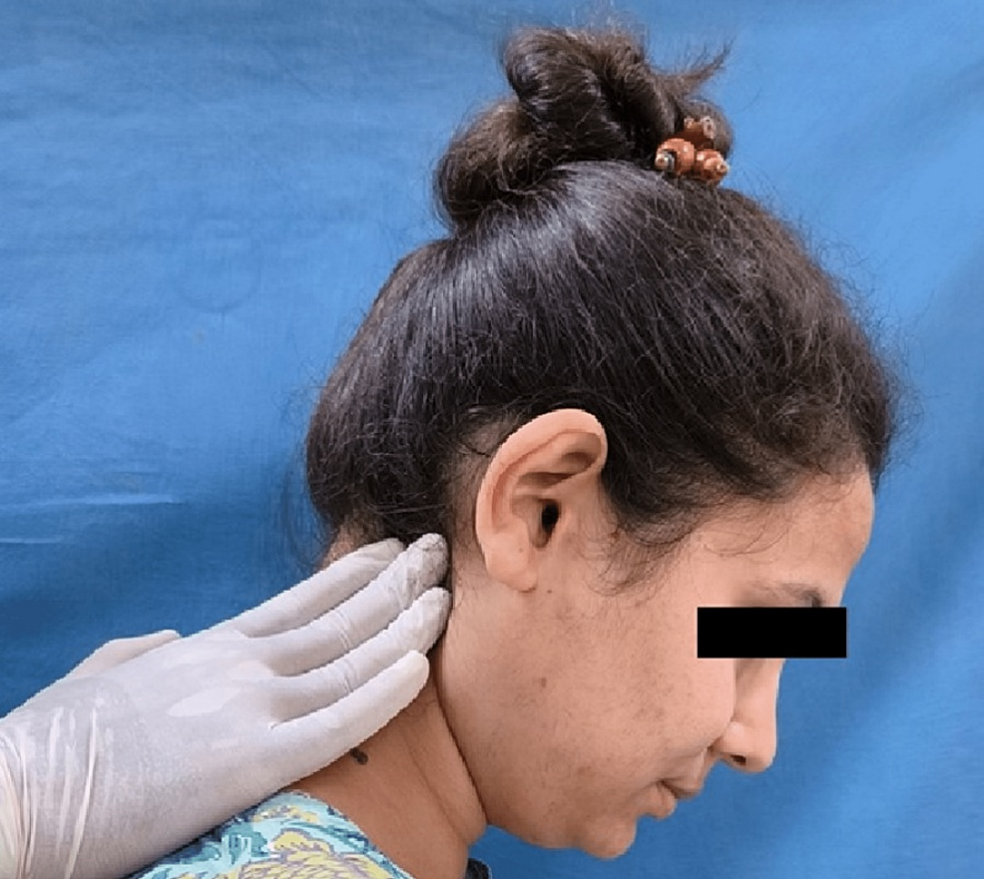
Palpation of the splenius capitis muscle

**Figure 4:**
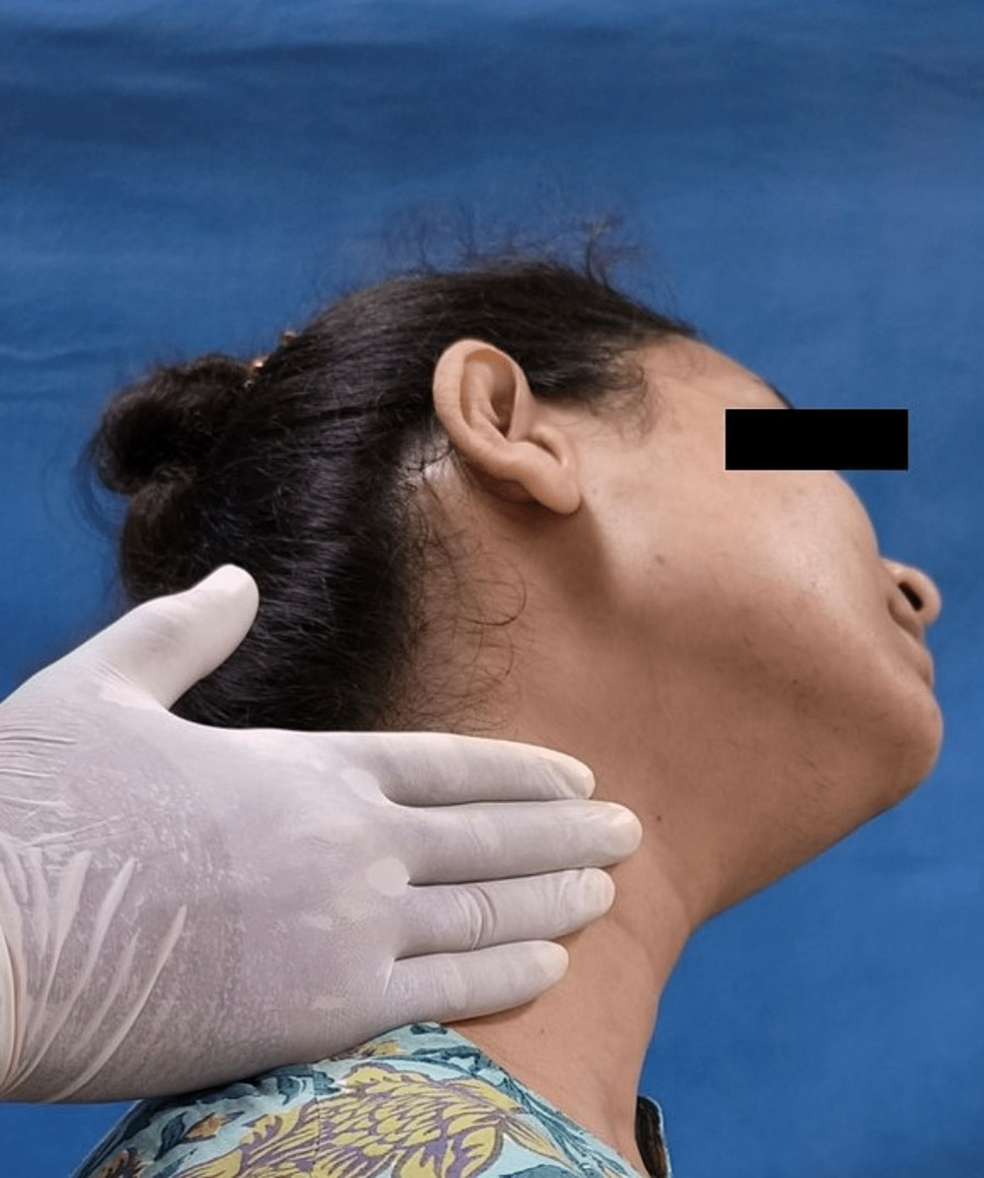
Palpation of the sternocleidomastoid muscle

**Figure 5:**
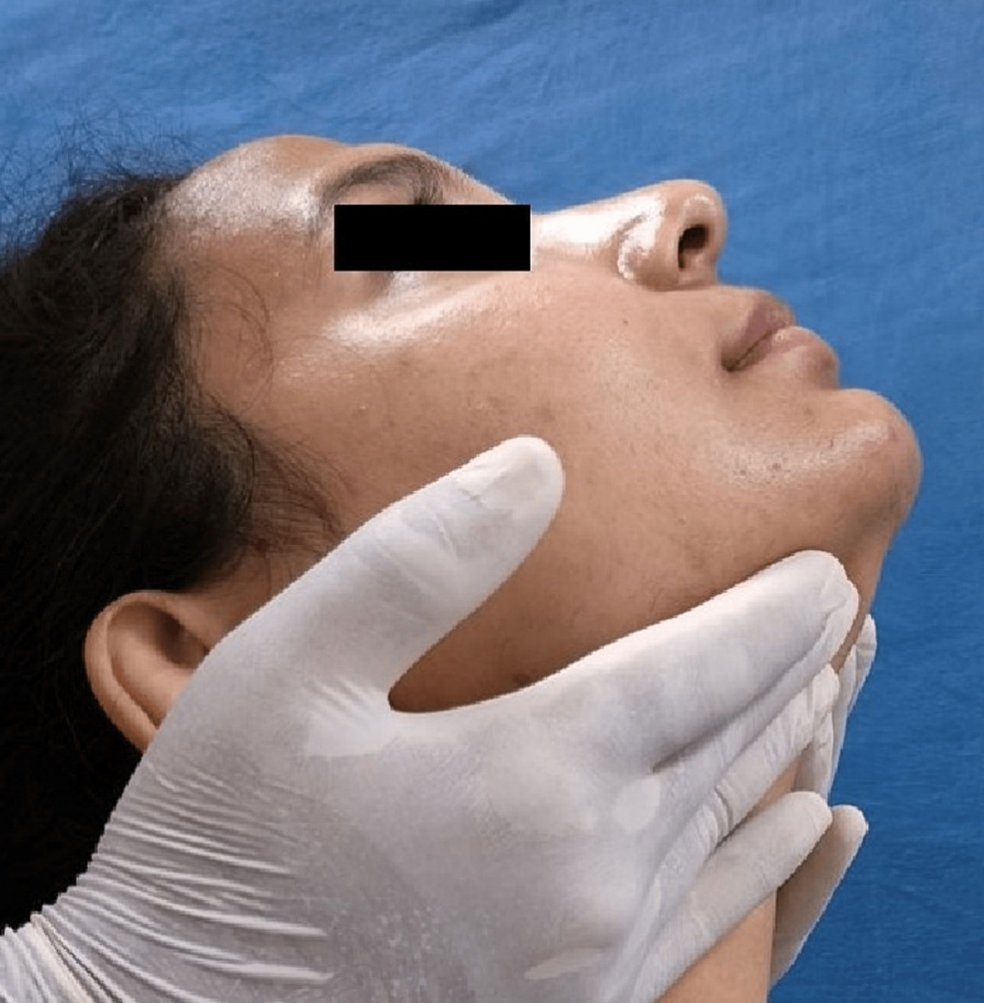
Palpation of the anterior digastric muscle

**Figure 6:**
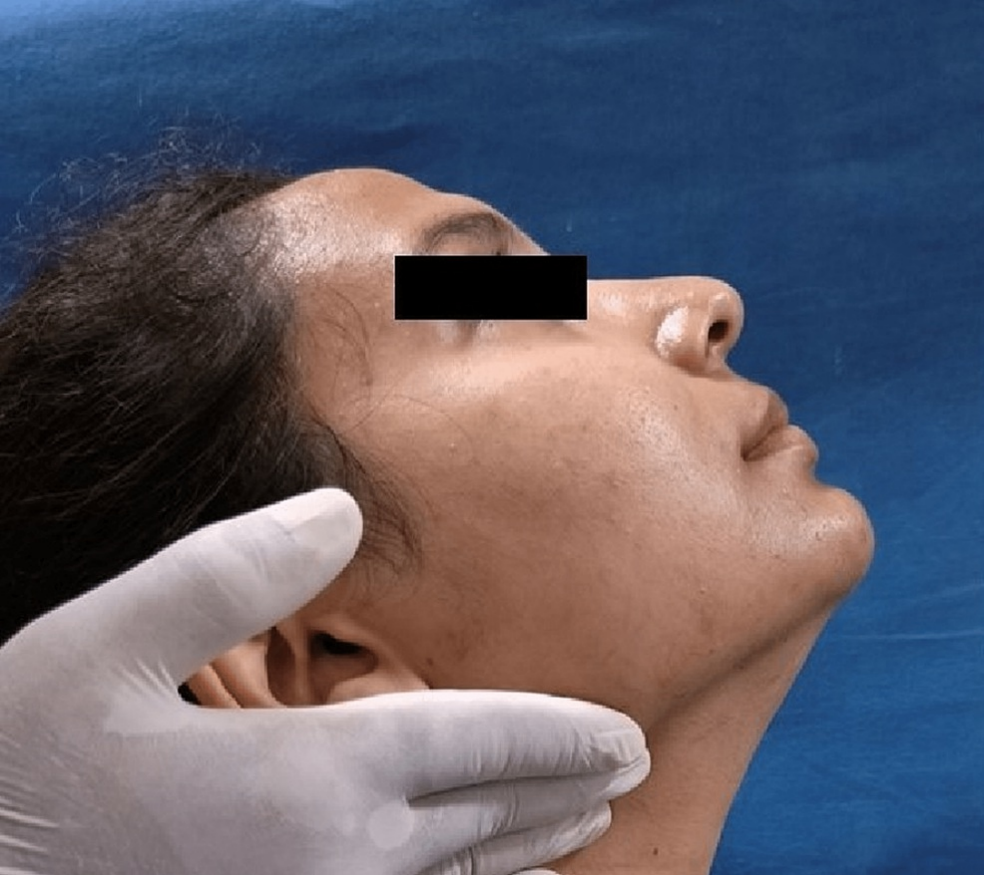
Palpation of the posterior digastric muscle

The Helkimo index graded patients' subjective and objective symptoms into no, mild, moderate, and severe categories. The neck musculature in all patients was evaluated for pain patterns using manual palpation with sustaining firm finger pressure along the muscle length. The trapezius (Figure 2), splenius capitis (Figure 3), sternocleidomastoid (Figure 4), anterior digastric (Figure 5), and posterior digastric muscle (Figure 6) were palpated bilaterally. The presence or absence of pain was recorded. For each muscle segment with the presence of pain, a score of 1 was assigned, and for the absence of pain score of 0 was assigned. Accordingly, five groups of muscles were considered (Table 1). This was followed by grading overall neck pain out of 10. A score of 0 indicated no neck pain, 1-3 indicated mild pain, 4-6 indicated moderate pain, and 7 or above indicated severe neck pain (Table 2). The pain in the neck musculature was correlated with the severity of TMD, considering the subjective and objective symptoms separately. For observation, all universal precautions and infection control procedures were taken. Informed and written consent was obtained from all the patients.

**Table 1:** Scoring for pain in the neck musculature.

Muscle	Scoring for the absence of pain=0, presence of pain=1.	
	Right	Left
Trapezius	0-1	0-1
Sternocleidomastoid	0-1	0-1
Splenius capitis	0-1	0-1
Anterior digastric	0-1	0-1
Posterior digastric	0-1	0-1
Total out of 10	Minimum score=0,Maximum score=10	

**Table 2:** Grading of pain in the neck musculature

Grading of pain severity	
Score	Inference
0	No pain in neck muscles
1-3	Mild pain in neck muscles
4-6	Moderate pain in neck muscles
7 and above	Severe pain in neck muscles

## Results

Statistical analysis was done by descriptive and inferential statistics using the Chi-square test, the software used in the analysis was SPSS version 27.0 (IBM Corp., Armonk, NY, USA), and p<0.05 was considered as the level of significance which revealed a statistically significant association between the severity of pain in all five groups of neck muscles and severity of TMD. The severity of pain increased with the increasing severity of TMD.

TMD grading based on Helkimo index

The Helkimo index consists of two components- the amnestic component (subjective symptoms) and the clinical dysfunctional component (objective symptoms). The grading results were as follows: out of 44 cases included, considering the objective symptoms, 11 cases (25%) each had no TMD symptoms, mild TMD symptoms, moderate TMD symptoms, and severe TMD symptoms, respectively. While out of the total 44 cases included, 10 cases (22.73%) had no subjective symptoms, 20 cases (45.45%) had mild subjective symptoms, and 14 cases (31.82%) had severe subjective symptoms (Table 3).

**Table 3:** Grading of temporomandibular disorder patients according to severity, based on the Helkimo index

Symptom severity	Number of patients in each group	
	Dysfunctional component	Amnestic component
No symptoms	11	10
Mild symptoms	11	20
Moderate symptoms	11	-
Severe symptoms	11	14

Correlation between pain in the neck musculature and severity of subjective TMD symptoms (clinical amnestic component)

**Figure 7:**
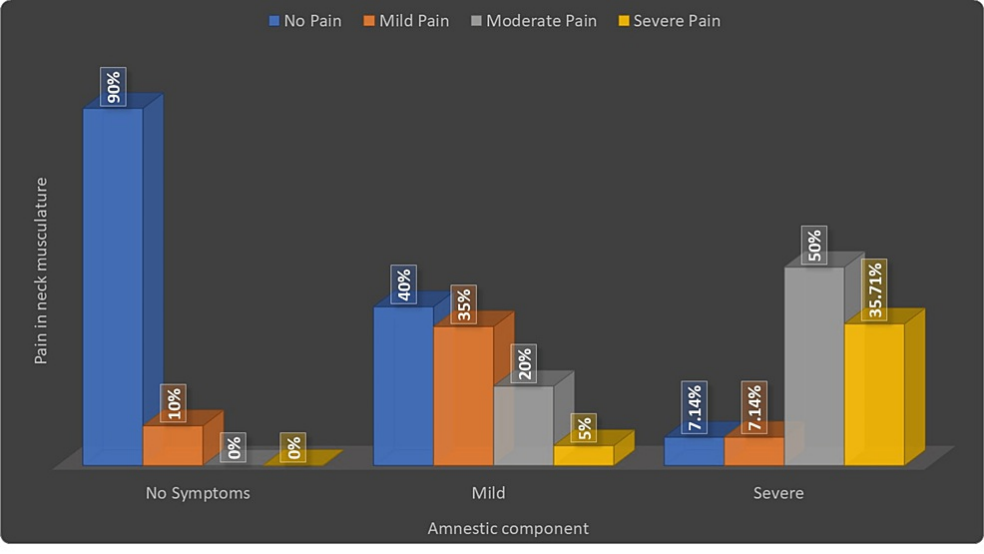
Correlation between pain in the neck musculature and clinical amnestic component

Out of 14 TMD patients with severe subjective symptoms, five patients (35.71%) had severe pain in the neck musculature, seven patients (50%) had moderate pain in the neck musculature, and one patient (7.14%) each had mild or no pain in the neck musculature respectively. Out of 20 patients who had mild subjective symptoms, one patient (5%) had severe pain, four patients (20%) had moderate pain, seven patients (50%) had mild pain, and eight patients (40%) had no pain in neck musculature. Out of 10 patients with no subjective symptoms, no patients had severe or moderate pain in the neck musculature. In comparison, one patient (10%) had mild neck pain, and 10 patients (90%) reported no pain in neck musculature (Table 4, Figure 7).

**Table 4:** Correlation between pain in the neck musculature and clinical amnestic component

Pain in the neck musculature	Amnestic component			Total
	No Symptoms	Mild	Severe	
No Pain	9(90%)	8(40%)	1(7.14%)	18(40.91%)
Mild Pain	1(10%)	7(35%)	1(7.14%)	9(20.45%)
Moderate Pain	0(0%)	4(20%)	7(50%)	11(25%)
Severe Pain	0(0%)	1(5%)	5(35.71%)	6(13.64%)
Total	10(22.73%)	20(45.45%)	14(31.82%)	44(100%)
ϗ2-value	27.27, p-value=0.0001, Significant			

Correlation between pain in the neck musculature and objective TMD symptoms (clinical dysfunctional component)

**Figure 8:**
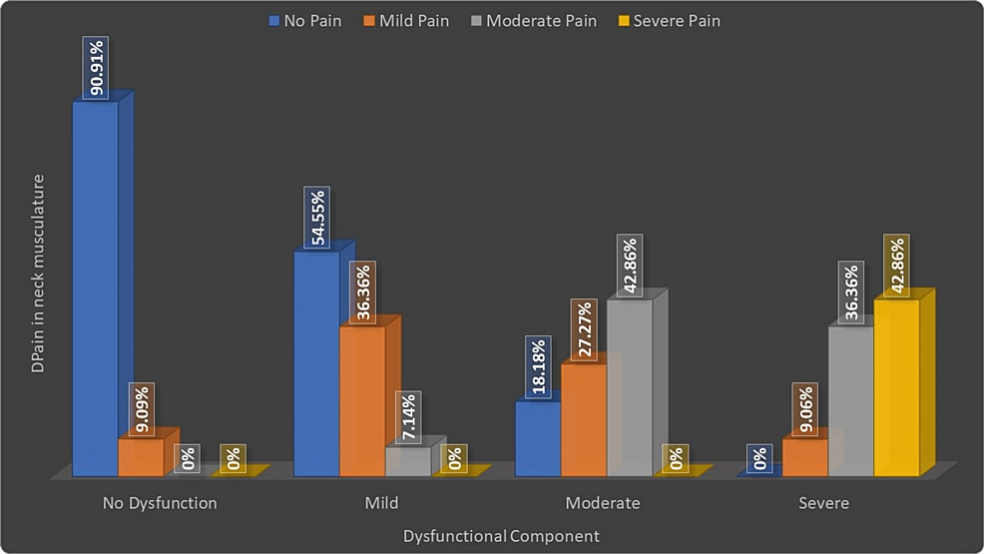
Correlation between pain in the neck musculature and clinical dysfunctional component

Out of the 11 TMD patients, considering the objective symptoms, who had severe dysfunction, six patients (42.86%) had severe neck pain, four patients (36.36%) had moderate, one patient (9.06%) had mild pain, and one had no pain. Out of the 11 patients with moderate dysfunction, no patients had severe neck pain, six patients (42.86%) had moderate neck pain, three patients (27.27%), and two patients (18.18%) had mild or no neck pain, respectively. Out of 11 patients with mild dysfunction, no patient had severe neck pain, one patient (7.14%) had moderate neck pain, four patients (36.36%) had mild pain, and six patients (54.55%) had no neck pain. Among 11 patients with no dysfunction, 10 patients (90.91%) had no neck pain, while one patient (9.09%) had mild neck pain (Table 5, Figure 8).

**Table 5:** Correlation between pain in the neck musculature and clinical dysfunctional component

Pain in the neck musculature	Dysfunctional Component				Total
	No Dysfunction	Mild	Moderate	Severe	
No Pain	10(90.91%)	6(54.55%)	2(18.18%)	0(0%)	18(40.91%)
Mild Pain	1(9.09%)	4(36.36%)	3(27.27%)	1(9.06%)	9(20.45%)
Moderate Pain	0(0%)	1(7.14%)	6(42.86%)	4(36.36%)	11(25%)
Severe Pain	0(0%)	0(0%)	0(0%)	6(42.86%)	6(13.64%)
Total	11(25%)	11(25%)	11(25%)	11(25%)	44(100%)
ϗ2-value	42.68, p-value=0.0001, Significant				

Amongst the 44 TMD cases, considering the subjective and objective symptoms,40.91% of patients (18 patients) had no pain in the neck musculature, 20.45% of patients (nine patients) had mild pain, 25% of patients (11 patients) had moderate pain, and 13.64% of patients (six patients) had severe pain, showing that a total of 59.09% of TMD patients presented with varying degrees of pain in the neck musculature with a p-value of 0.0001% which was significant.

## Discussion

The present study was aimed at correlating the severity of neck pain with the severity of TMD. Firstly, to grade the TMD patients according to severity, the Helkimo index was used, which consists of an amnestic component (assessing subjective symptoms) and a dysfunctional component (assessing objective symptoms). The amnestic component is based on a questionnaire that includes questions relating to TMJ sounds, jaw rigidity, and pain in the masticatory muscles, among others. The dysfunctional component includes a clinical examination of the patient examining mandibular movements, palpating masticatory muscles, and assessing clicking and luxation. The final grading is based on dysfunction inferred depending on a 25-point scale. Both components were correlated with neck pain severity [12]. Associating subjective and objective symptoms with pain in neck musculature was necessary as early TMD may present with only subjective symptoms. However, the pain in neck muscles may still be present and give an early diagnosis of TMD [13].

Symptoms of TMD include TMJ pain, clicks or crepitus, headache, myofascial pain, reduced mandibular range of motion, masticatory muscle exhaustion, and the constraint of mouth opening, which are assessed routinely [14]. However, some extra craniofacial symptoms like pain in the neck musculature, neural symptoms, and ear-related symptoms may also be present in patients with TMD, which may be an early sign of developing TMD [15,16]. Previously a study done by Bargatto et al., assessing the co-existence of neck pain in office workers, concluded that neck muscle pain correlated with TMD but computer-based work activity was an aggravating factor [17]. This can be correlated with our finding of neck muscle pain in 10% of the non-TMD cases, which may be due to factors other than TMD. However, there are very few studies directly correlating pain in the neck muscles with the severity of TMD. One such study was done by Wright, which evaluated referred craniofacial pain in patients with TMD's and concluded that 85% of cases had referred craniofacial pain generated on palpation of muscles of which trapezius was one of the most involved muscles [3]. Our study evaluated the trapezius muscle and other neck muscles, such as the sternocleidomastoid, anterior and posterior digastric muscles, and splenius capitis muscle bilaterally, which could detect overall neck pain severity in line with the craniomandibular index [18].

This study observed that the severity of neck pain increased as the severity of TMD increased. Most cases that reported mild to moderate subjective or objective TMD symptoms had mild to moderate pain in the neck musculature. One patient with mild subjective symptoms reported severe neck pain, which would require examination for additional non-TMD related aggravating factors such as anxiety or genetics, as reported by Yalcinkaya et al. and Fejer et al., respectively, in separate studies which concluded that neck pain may be aggravated due to anxiety or may be inherited [19,20]. Patients with severe subjective TMD symptoms reported varying severity of neck muscle pain, but more than 50% of patients had moderate to severe pain. This finding was in line with the findings of Olivo, who concluded that cases with a higher grade of jaw disability had a higher grade of a neck disability, too [21]. These findings confirm that though not routinely examined, pain in the neck muscles may be a co-existing symptom in patients with TMJ dysfunction and can give an early insight into the diagnosis and severity of TMD. Manual palpation of neck muscles, one of the simplest assessment methods [22], if regularly done while examining TMD cases, can be of prime diagnostic importance and pave the way for early intervention for managing TMD [23]. Since no study was done in the past correlating symptom-based severity of neck pain with TMD severity, this study becomes a basis for laying down a standardized diagnostic protocol for evaluating TMD.

Limitations of this study include that no radiographic assessment like MRI was done while clinically assessing TMD. Combining both clinical and radiographic evaluation would give a definitive diagnosis and a better correlation with pain in the neck musculature. Once a detailed diagnosis of TMD is obtained based on the craniofacial and extra-craniofacial symptoms, appropriate management protocol can be followed. Based on the severity of TMD, a pharmacological or non-pharmacological approach can be opted for. Pharmacological approaches include the use of analgesics for symptomatic relief or use of muscle relaxants in patients reporting with pain in the jaw or neck musculature. Nonpharmacological approaches include occlusal splints, physiotherapy, and transcutaneous electrical nerve stimulation, among others. Severe TMD cases may require further surgical intervention.

## Conclusions

From the results of this study, it was concluded that neck muscle pain co-existed with TMD in the majority of the cases, and the severity of TMD had a direct correlation with pain in various groups of neck muscles such as the splenius capitis, trapezius, sternocleidomastoid, and the anterior and posterior digastric muscles. The severity and distribution of pain in the neck muscles increased with the increasing severity of TMD. At the same time, non-TMD cases had minimal pain in the neck muscles. Not only the objective symptoms but also the subjective symptoms had a direct correlation with TMD, which also harmed the social and personal lives of patients. Neck pain can be considered an initial symptom of a developing TMD. Pain in the neck musculature can be of utmost diagnostic importance. It can be helpful in early screening, diagnosis, and planning treatment for patients with temporomandibular dysfunction, paving the way for identifying undiagnosed cases and early intervention in such cases to reduce morbidity. Adding neck muscle evaluation in the diagnostic protocol of TMD would lead to a better quality of life in such patients.
